# Pleiotropic Roles of Scavenger Receptors in Circadian Retinal Phagocytosis: A New Function for Lysosomal SR-B2/LIMP-2 at the RPE Cell Surface

**DOI:** 10.3390/ijms23073445

**Published:** 2022-03-22

**Authors:** Quentin Rieu, Antoine Bougoüin, Yvrick Zagar, Jonathan Chatagnon, Abdallah Hamieh, Julie Enderlin, Thierry Huby, Emeline F. Nandrot

**Affiliations:** 1 Sorbonne Université, INSERM, CNRS, Institut de la Vision, 17 rue Moreau, F-75012 Paris, France; quentin.rieu@inserm.fr (Q.R.); antoine.bougouin@crc.jussieu.fr (A.B.); yvrick.zagar@inserm.fr (Y.Z.); jchatagnon@gmail.com (J.C.); hamieh.abdallah@gmail.com (A.H.); julie.enderlin@inserm.fr (J.E.); 2 Sorbonne Université, INSERM, UMR-S 1166, F-75013 Paris, France; thierry.huby@sorbonne-universite.fr

**Keywords:** retinal pigment epithelium, phagocytosis, circadian function, scavenger receptors, class A, class B, SR-B2/LIMP-2, MARCO

## Abstract

The retinal phagocytic machinery resembles the one used by macrophages to clear apoptotic cells. However, in the retina, the permanent contact between photoreceptor outer segments (POS) and retinal pigment epithelial (RPE) cells requires a tight control of this circadian machinery. In addition to the known receptors synchronizing POS internalization, several others are expressed by RPE cells. Notably, scavenger receptor CD36 has been shown to intervene in the internalization speed. We thus investigated members of the scavenger receptor family class A SR-AI and MARCO and class B CD36, SR-BI and SR-B2/LIMP-2 using immunoblotting, immunohisto- and immunocytochemistry, lipid raft flotation gradients, phagocytosis assays after siRNA/antibody inhibition, RT-qPCR and western blot analysis along the light:dark cycle. All receptors were expressed by RPE cell lines and tissues and colocalized with POS, except SR-BI. All receptors were associated with lipid rafts, and even more upon POS challenge. SR-B2/LIMP-2 inhibition suggested a role in the control of the internalization speed similar to CD36. In vivo, MARCO and CD36 displayed rhythmic gene and protein expression patterns concomitant with the phagocytic peak. Taken together, our results indicate that CD36 and SR-B2/LIMP-2 play a direct regulatory role in POS phagocytosis dynamics, while the others such as MARCO might participate in POS clearance by RPE cells either as co-receptors or via an indirect process.

## 1. Introduction

Phagocytic cells use several types of receptors expressed at their cell membrane in order to fulfill their role in efficiently eliminating apoptotic cells (AC), bacteria, and viruses. Two categories of phagocytes exist, namely professional and non-professional, such as macrophages and cells from the retinal pigment epithelium (RPE), respectively. One of the main roles of RPE cells is the daily phagocytosis of aged tips of photoreceptor outer segments (POS) that are constantly renewed [1]. Each RPE cell serves around 25 POS and, RPE cells being post-mitotic, they are actually the busiest phagocytes in the body. An important feature of retinal phagocytosis is the daily rhythmic variation of its activity [2]. Importantly, any defect in its timely completion leads to the development of blinding diseases in animal models [3,4,5] and in patients [6,7]. Indeed, when phagocytosis does not occur, or is severely impaired, early-onset rod-cone dystrophies (RCD) take place [3,4,6,7]. Alternatively, if POS phagocytosis is arrhythmic, a late-onset cumulative phenotype develops with loss of vision and lipofuscin accumulation, typical features of age-related macular degeneration (AMD) [5]. Photoreceptors and RPE are closely interdependent cells, in particular because photoreceptors do not have direct access to the blood stream and rely on RPE cells. Thus, phagocytosis RPE cells support several functions, all crucial for photoreceptor survival, and hence maintenance of vision such as light absorption, recycling of visual cycle molecules, secretion of trophic factors, and the bilateral transport of water, ions and vitamins [1].

The phagocytic molecular machinery at the surface of RPE cells resembles the one used by macrophages to eliminate AC [8], and is sequentially organized. We have previously shown that POS phagocytosis is synchronized via the αvβ5 integrin receptor and its ligand MFG-E8 [5,9]. We also identified MerTK as fulfilling two functions, regulating the amounts of POS that can be bound by RPE cells [10], and being required for their subsequent internalization [4]. The tethering of both AC and POS is triggered via the recognition of phosphatidylserines (PtdSer), “eat-me” signals exposed on their external membrane leaflet [11,12]. PtdSer can be bound either directly by receptors expressed at the phagocyte cell surface, or indirectly via several ligands in the extracellular space that act as bridging molecules. Indeed, MFG-E8 and Gas6/Protein S, ligands for αvβ5 integrin and MerTK receptors respectively, recognize PtdSer and contribute to the regulation of POS phagocytosis [9,13,14,15].

Unlike macrophages, RPE cells do not need the “find me” signal priming because of the permanent contact between POS and RPE cells. However, RPE cells only phagocytose once a day. Thus, phagocytosis by RPE cells has to be highly controlled via multiple pathways, notably under the impulse of the αvβ5 integrin/MFG-E8 couple as the daily launch signal [9]. Indeed, we recently showed that a soluble version of the MerTK receptor produced by the cleavage of its whole extracellular domain regulates POS phagocytosis by acting as a decoy receptor [15]. In addition, and also in contrast with macrophages, MerTK ligands Gas6 and Protein S appear to be bear opposite roles in the retina, Gas6 acting as an inhibitor and ProteinS as a stimulator [15]. Our most recent results suggest that respective Gas6 and ProteinS levels available in the interphotoreceptor matrix could contribute to regulating MerTK activity by a competitive action, with these ligands recognizing slightly different amino acids on MerTK Ig-like domains (Parinot et al., under revision).

Each receptor or receptor/ligand couple of the phagocytic machinery accomplishes a specific role during the phagocytic process. While the respective roles of αvβ5 integrin and MerTK receptors are known, the potential participation of other receptors or co-receptors has been shown or suggested by various studies in different tissues. Indeed, the CD81 tetraspanin acts as an accessory receptor for the αvβ5 integrin to promote POS binding to RPE cells [16]. Besides integrins and TAM receptors (Tyro3, Axl, MerTK), many other receptors are able to target PtdSer, either directly or indirectly, such as lectins, receptors recognizing complement molecules and low-density lipoproteins (LDLs), CD14, and scavenger receptors (SR) [17]. According to the latest nomenclature, SRs are divided in 11 different classes of pattern recognition receptors, ranging from A to L, most of them being expressed in various types of macrophages [18,19,20]. Class B SRs CD36 (cluster of differentiation 36, SCARB3) and SR-BI/II (scavenger receptor class B type I or II isoforms, SCARB1, CD36L1), as well as endosomal class D SR CD68 (macrosialin) and class E SR LOX1 (lectin-like oxidised LDL receptor-1) can bind PtdSer directly [21,22,23,24].

Previously, CD36 receptors have been shown to be implicated in POS elimination via direct PtdSer binding on the apical side of RPE cells [21,25]. Indeed, CD36 appears to regulate the speed of POS uptake after they are bound to the cells [26], and CD36 expression is altered in RCS rat RPE devoid of MerTK that does not internalize POS [27]. Moreover, CD36 phagocytosis seems to be augmented in stress conditions with oxidized POS or with lipids in AMD eyes [28,29], consistent with the observation that CD36 only binds with oxidized PtdSer in macrophages [30]. CD36 appears to lead the intake of oxidized LDL at the Bruch’s membrane level in association with sub-retinal deposits on the RPE basal side, suggesting CD36 receptors bear pleiotropic functions in these cells [31].

RPE cells readily express other receptors from the SR family, such as class A MARCO (macrophage receptor with collagenous structure, SCARA2) [32] and class B SR-BI [33,34,35], but their exact participation in POS phagocytosis has not been investigated yet. Of note, the basal transport of POS-derived lipids by RPE cells was abolished by a SR-BI inhibitor, glyburide, in the presence of high-density lipoproteins [35]. In addition, *SR-BI^+/−^* mice displayed a thickened Bruch’s membrane, while in *SR-BI* knockout mice subRPE deposits, retina/RPE morphological and functional alterations were detected after a long-term atherogenic diet in addition to atherosclerotic plaques detected in the heart [35,36]. Interestingly, MARCO has been recently shown to bind the αvβ5 integrin receptor and activate cell signaling, promoting pseudopodia numbers in macrophages [37].

On a similar note, the class A SR-AI (macrophage scavenger receptor 1 MSR1, SCARA1, CD204) SR is involved in the clearance of apoptotic cells by macrophages and signals via the MerTK internalization receptor [38,39]. Coincidentally, the MerTK ligand Gas6 seems to induce SR-AI expression in muscle cells [40]. SR-A receptors recognize various ligands, including chemically modified molecules such as PtdSer-expressing apoptotic cells and stress-related advanced glycation end products (AGE) [41]. However, SR-AI knockout mice did not display any apoptotic cell elimination defect, thus suggesting that it might act as an accessory receptor [42].

Less studied, class B SR-B2/LIMP-2 (lysosomal integral membrane protein 2, LIMPII, SCARB2, CD36L2) is a receptor involved in lysosomal cholesterol export with a 3D structure similar to CD36 and SR-BI [43,44]. Interestingly, SR-B2/LIMP-2 seems to play a role in macrophage activation and late phagosomal trafficking [45]. In addition, SR-B2/LIMP-2 deficiency has been shown to be associated with peripheral neuropathy in mice [46].

The potential implication of class A and B SRs in the regulation of POS phagocytosis, besides CD36, has not been explored so far. Hence, we set out to characterize the expression levels in cultured cells and retinal tissues as well as the colocalization with POS and signaling membrane subdomains of class A SR-AI and MARCO and class B CD36, SR-BI and SR-B2/LIMP-2. We also assessed the effect of their downregulation on POS phagocytosis and their circadian expression profile in vivo.

## 2. Results

### 2.1. All Receptors Are Readily Expressed by RPE Cells and Colocalize with POS except SR-BI

**Figure 1:**
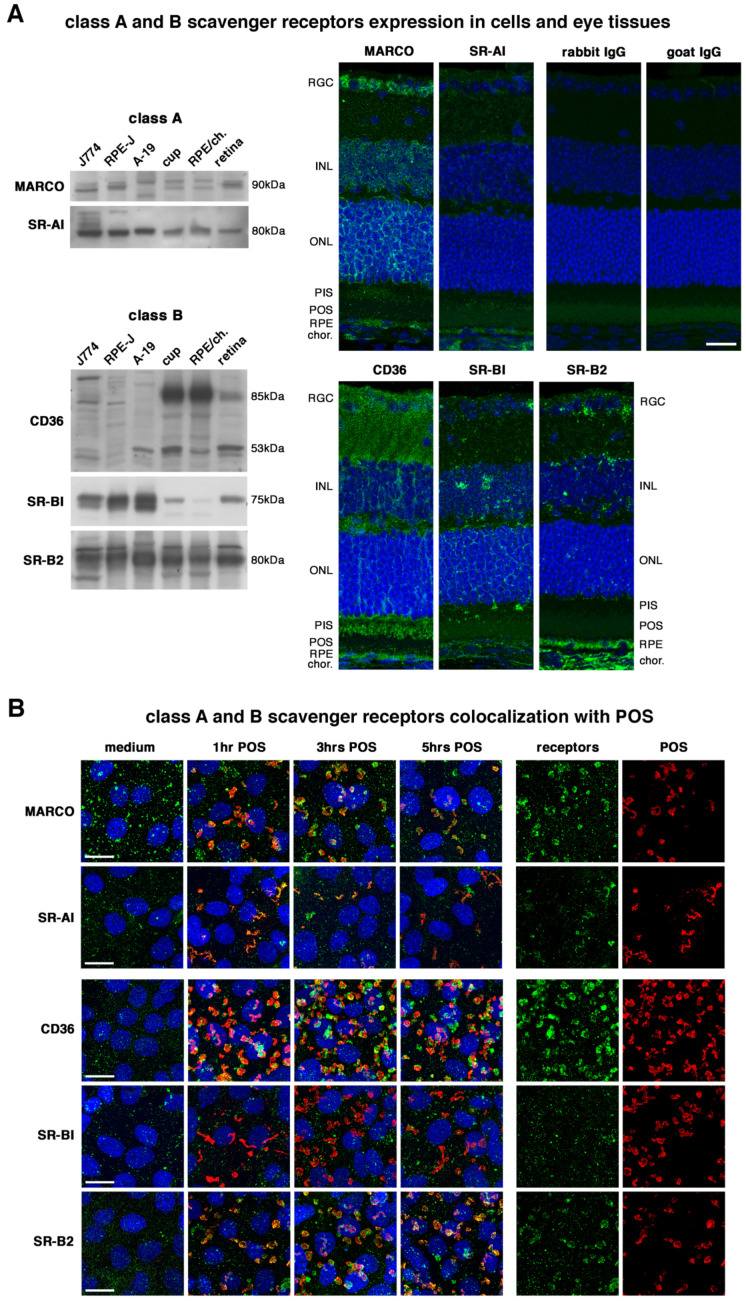
All receptors are expressed by RPE cells and all except SR-BI colocalize with POS. Representative immunoblots (**A**) show that class A scavenger receptors MARCO and SR-AI (left) and class B CD36, SR-BI and SR-B2/LIMP-2 (right) are all expressed in J774.1 macrophages (J774), rat RPE-J, and human ARPE-19 (A-19) as well as in dissected eyecups (cup), and separated RPE/choroid (RPE/ch.) and retina as indicated. MARCO and CD36 receptors show size differences between tissues and species. Immunohistochemistry labelings on paraffin tissue sections (**A**) confirm the high abundance of CD36 and SR-B2/LIMP-2, and the RPE expression of all 5 receptors. RGC: retinal ganglion cells; INL: inner nuclear layer; ONL: outer nuclear layer; PIS: photoreceptor inner segments; POS: photoreceptor outer segments; RPE: retinal pigment epithelium; chor.: choroid. Immunocytochemistry labelings (**B**) show that all receptors (green) colocalize with POS (red) except SR-BI after 1 (1 hr), 3 (3 hrs) and 5 (5 hrs) hours of POS challenge when compared to medium incubation, as indicated. Nuclei are in blue. The 2 panels on the right correspond to separate channels for receptors (green) and POS (red) for the time-point with maximum colocalization (MARCO 3 hrs, SR-AI 1 hr, CD36 3 hrs, SR-B2/LIMP-2 1 hr) or surface receptor expression (SR-BI 3 hrs). Scale bars: 20 µm.

We assessed the respective expression profiles of our five candidates in cultivated J774.1 macrophages, rat RPE-J, and human ARPE-19 cells, as well as in murine full eyecups and separated RPE/choroid and retina tissues. Class A SRs MARCO and SR-AI were expressed at equivalent levels in all samples with some slight molecular weight differences between cell types and tissues (Figure 1A). On eye paraffin sections, MARCO was expressed in several retinal cell types, with an emphasis on the RPE and retinal ganglion cell (RGC) layers, while SR-AI highest expression levels were detected in the RPE (Figure 1A). At the cellular level, MARCO receptors showed a punctate pattern at the RPE-J cell surface in native conditions (Figure 1B). Upon POS challenge, MARCO receptors colocalized with POS after 1, 3, and 5 hours of incubation, with a colocalization peak reached at 3 hours (separated channels on Figure 1B). In contrast, SR-AI receptors displayed a weaker surface expression pattern that seemed to increase upon POS challenge with a colocalization maximum at 1 hour. All medium condition time-points gave similar signals for all receptors, and only one time-point was shown for the control reference (3 hours).

Class B SRs, CD36 and SR-BI, showed more or less pronounced expression in in vivo tissues when compared with cultured cells, respectively (Figure 1A). In contrast, SR-B2/LIPM-2 Class B SR was similarly expressed at high levels both in vitro and in vivo. Of note, CD36 receptors were highly expressed in retinal tissues when compared to macrophages or RPE cell lines. Tissue sections confirmed the high levels of CD36 expression throughout the retina (Figure 1A). SR-BI and SR-B2/LIMP-2 expression were more restricted, mostly to the RPE and blood vessels in the retina and the choroid. In vitro, all three class B SRs were weakly expressed at the cell surface in unstimulated cells (Figure 1B). CD36 surface expression was greatly increased in cells incubated with POS, a phenomenon also present for SR-B2-LIMP-2, but in a more limited fashion. Maximum colocalization of both receptors with POS was observed at 3 and 1 hours, respectively. No such surface expression increase or POS colocalization was detected for SR-BI receptors.

### 2.2. All Receptors Associate with Lipid Rafts

**Figure 2:**
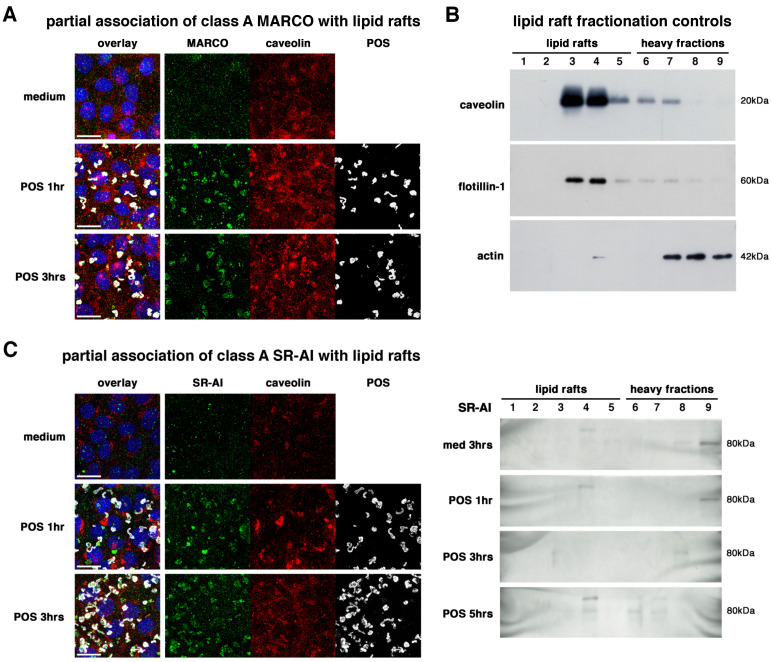
Partial association of class A scavenger receptors with lipid rafts. Immunocytochemistry labelings (**A**,**C**) show that MARCO (**A**) and SR-AI (**C**) receptors (green) partially associate with lipid rafts marker caveolin (red) after 1 (POS 1 hr) and 3 (POS 3 hrs) hours of POS (grey) challenge when compared to medium incubation as indicated. Overlay panels (left) include nuclei (blue). Scale bars: 20 µm. Immunoblots of flotation gradient (**B**,**C**) fractions (1 to 9) show the proper biochemical fractionation of cells separating lipid rafts (fractions 1–5) from heavier cell parts (fractions 6–9) as assessed by specific lipid rafts markers caveolin (top) and flotillin-1 (middle), and cell cytoskeleton marker actin (bottom), as indicated (**B**). SR-AI-specific immunoblots confirm the partial association of POS and receptors to lipid rafts, especially at 3 and 5 hours (POS 3 hrs, POS 5 hrs) when compared to a 3-hour incubation with medium (med. 3 hrs), as indicated (**C**).

Surface expression of most candidates showed punctate patterns in cultured RPE-J cells, suggesting the presence of receptor clusters (Figure 1B). Using flotation gradients, we thus investigated the association of SRs with lipid rafts, lipid-enriched cell surface microdomains known to serve as signaling platforms by associating cytoskeletal proteins and transduction pathways, thus allowing the cells to respond efficiently to extracellular signals [47,48]. As for POS colocalization assays, with all medium conditions being similar, only one time-point (3 hours) was shown. MARCO receptors displayed the largest cluster sizes but were only partially associated with lipid raft marker caveolin observed by immunocytochemistry (ICC), especially after 1 hour of POS challenge (Figure 2A). Unfortunately, our biochemistry confirmation studies did not allow us to detect any signal with the three different antibodies (data not shown). We suspect that both the dilution factor generated by the gradient fractionation combined with the limited MARCO expression level detected by western blots in RPE cells (Figure 1) gave insufficient signals. We confirmed that our fractionation protocol allowed for the proper separation of “floating” lipid rafts detected by caveolin and flotillin-1 markers (concentrated in lanes 3 and 4, with more limited signals in lanes 5 to 7), from heavier fractions containing other markers such as actin (lanes 7 to 9) (Figure 2B). Similarly to MARCO, SR-AI receptors were partially associated with lipid rafts after POS challenge both in ICC and biochemistry fractionation experiments (Figure 2C). This association increased with time of phagocytosis, mostly at 3 and 5 hours of phagocytosis.

**Figure 3:**
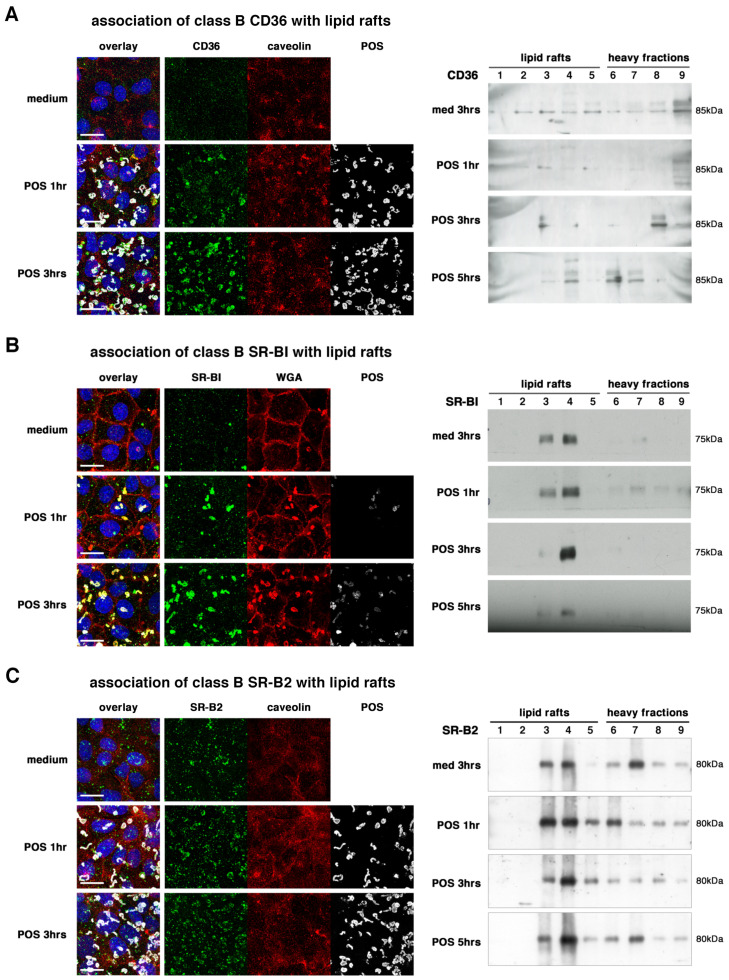
Association of class B scavenger receptors with lipid rafts. Immunocytochemistry labelings (**A**–**C**) show that CD36 (**A**) and SR-B2/LIMP-2 (**C**) receptors (green) associate with lipid rafts marker caveolin (red) (**A**,**C**) after 1 (POS 1 hr) and 3 (POS 3 hrs) hours of POS (grey) challenge when compared to medium incubation as indicated. Overlay panels (left) include nuclei (blue). Scale bars: 20 µm. SR-BI shows a strong association with N-acetyl glucosamine (GlcNAc)-related oligosaccharides marker WGA present on cell surface-expressed proteins (**B**). Immunoblots of flotation gradients (**A**–**C**) confirmed that CD36 (**A**), SR-B2/LIMP-2 (**C**), and to a lower extent SR-BI (**B**) relocalize to membrane fractions corresponding to lipid rafts (1–5) from heavier fractions (6–9) at 3 and 5 hours (POS 3 hrs, POS 5 hrs) (CD36, (**A**); SR-BI, (**B**)) or at 3 hours only (SR-B2/LIMP-2, (**C**)) when compared to a 3 -hour incubation with medium (med. 3 hrs), as indicated.

Without stimulation, CD36 receptors were weakly expressed at the RPE cells surface and were present in all biochemistry subfractions (Figure 3A). Upon POS challenge, POS-bound CD36 SRs progressively colocalized with caveolin and were concentrated in lipid raft fractions after 3 and 5 hours. Interestingly, both SR-BI and SR-B2/LIMP-2 were mostly present in lipid rafts in the resting state (fractions 3–4 and 5–7), and were even more concentrated in lipid raft fractions 3–4 after 3 hours of POS incubation, and then returned to the unstimulated state distribution at 5 h (Figure 2B,C). Due to antibody species limitation, we were not able to verify the lipid raft association of SR-BI with lipid raft markers. We did analyse the colocalization of SR-BI with wheat germ agglutinin (WGA) lectins that specifically labeled N-acetyl glucosamine (GlcNAc)-related oligosaccharides on proteins expressed at the cell surface (Figure 3B) [49]. Interestingly, while colocalization of SR-BI with POS and WGA was extremely limited, SR-BI did strongly associate with WGA signals at 1 and even more at 3 hours, and those complexes appeared to make large clusters in areas of the cells not associated with POS. In contrast, POS-bound SR-B2/LIMP-2 receptors colocalized also with caveolin, especially at 3 hours when the concentration of the receptors in lipid rafts was also observed by biochemistry (Figure 3B).

### 2.3. Various Effects of Receptors Inhibition on POS Phagocytosis

**Figure 4:**
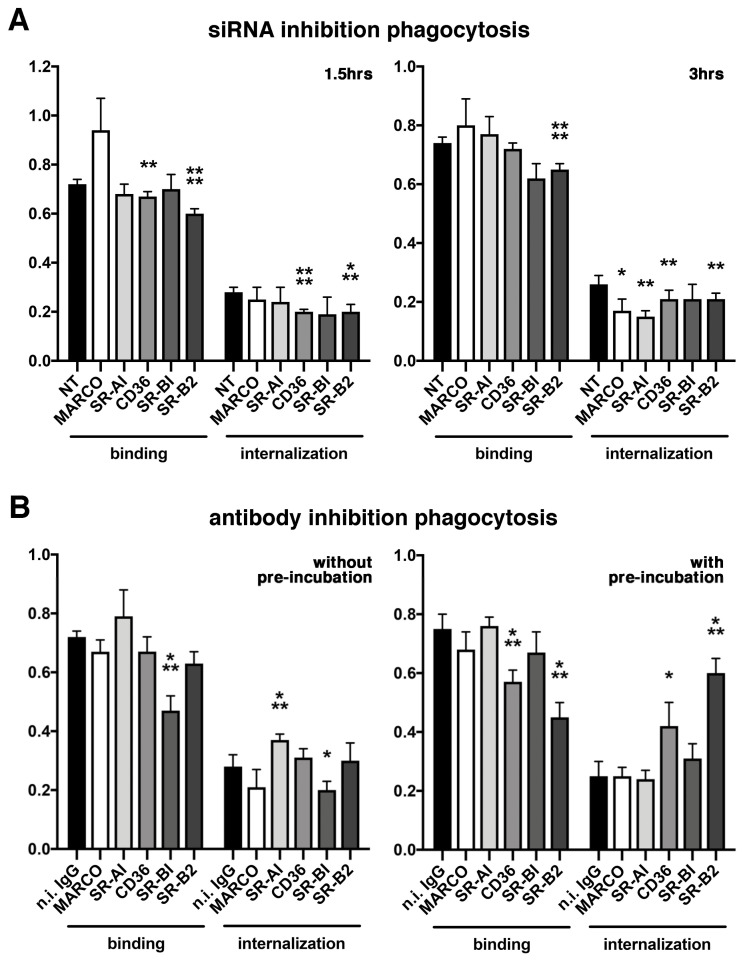
Various effects of receptors inhibition on POS phagocytosis. siRNA inhibition (**A**) assays show that class A MARCO and SR-AI downregulation only decreased POS internalization at 3 hours (right) but neither POS binding nor any phagocytosis step at 1.5 hours (left), as indicated. Class B *CD36* and *SR-B2*/*LIMP-2* downregulation decreased both POS binding and internalization at 1.5 hours, *CD36* internalization at 3 hours and *SR-B2*/*LIMP-2* binding and internalization at 3 hours. Downregulation of SR-BI had no effect on POS phagocytosis. NT: Non-targeting control. Antibody inhibition assays (**B**) show that blocking SR-AI increased POS internalization while blocking SR-BI decreased POS binding and internalization when antibodies were applied without pre-incubation (left bar graph). Pre-incubation (right bar graph) shows decreased POS binding and increased POS internalization when using anti-CD36 and SR-B2/LIMP-2 antibodies. Blocking antibodies against MARCO had no effect on phagocytosis. n.i. IgG: non-immune IgG controls. Mean ± s.d., statistical significance assessed via the Bonferroni–Dunn method for multiple comparisons versus the respective control (NT siRNA or non-immune IgG), * *p* < 0.05, ** *p* < 0.01, *** *p* < 0.001, **** *p* < 0.0001, *n* = 4–9 (**A**) and *n* = 3–6 (**B**).

Our next step was to assess the functional effect of the inhibition of these 5 SRs on POS phagocytosis by RPE cells, both at the mRNA and protein levels (Figure 4). siRNA inhibition assays showed only POS internalization at 3 hours was decreased for class A receptors MARCO (−35%) and SR-AI (−42%) (Figure 4A). Both class B CD36 and SR-B2/LIMP-2 downregulations significantly decreased POS binding (−7% and −16%, respectively) and internalization (−29% for both) after 1.5 hours of phagocytosis. After 3 hours, the same reduction in both phagocytosis steps was observed for SR-B2/LIMP-2 (−16% and −19%, respectively) while the inhibition of CD36 gene expression only affected POS internalization (−19%). No effect of SR-BI gene downregulation could be detected.

We proceeded with antibody inhibition assays with or without pre-incubation of the cells with the blocking antibody before a 3-hour phagocytic challenge (Figure 4B). This protocol allowed us to distinguish between receptors acting alone or requiring association with other receptors/proteins to fulfill their function [26]. Interestingly, these two conditions gave very different results: without pre-incubation, antibody blocking increased POS internalization of class A SR-AI (+32%) and decreased POS binding and internalization of class B SR-BI (−34% and −28%, respectively); pre-incubation of blocking antibodies impacted CD36 and SR-B2/LIMP-2 phagocytosis. Strikingly, for both of these class B receptors, binding was diminished (−24% and −40%, respectively) while at the same time internalization was greatly augmented (+68% and +140%, respectively), suggesting the acceleration of phagocytosis. However, the blocking of MARCO using antibodies did not have any impact on POS phagocytosis.

### 2.4. Class A MARCO and Class B CD36 Show Rhythmic Expression Patterns In Vivo

**Figure 5:**
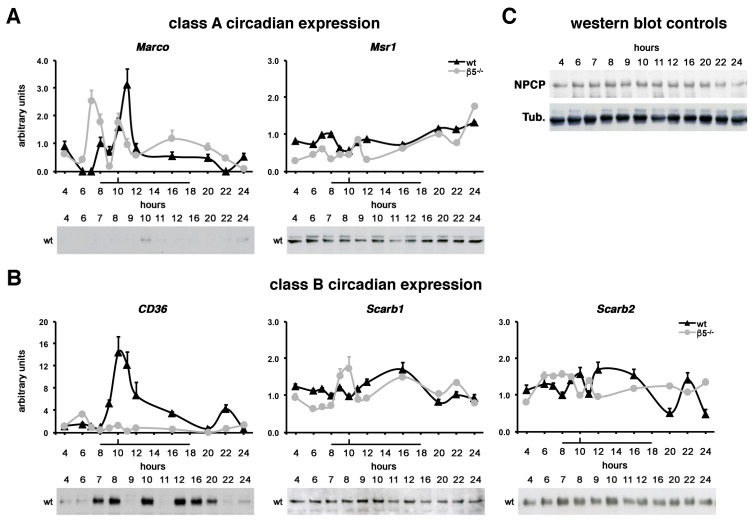
Class A MARCO and class B CD36 show rhythmic expression patterns in vivo. qPCR quantification of RPE/choroid gene expression and associated western blots of protein expression profiles (**A**,**B**) at different times along the light:dark cycle (horizontal bar indicates light) show that *Marco* (**A**) and *CD36* (**B**) displayed a peak of gene expression at 11.00 and 10.00–11.00 at or just after the phagocytic peak (black wedge) in wild-type controls (wt, black line and triangles) when compared to arrhythmic RPE/choroid from integrin β5 knockout mice (β5^−/−^, grey line and dots) as indicated. *Scarb1* (SR-BI) gene expression displayed a slight peak at 9.00–10.00 in *β5*^−/−^ samples (**B**), and *Scarb2* (SR-B2/LIMP-2) decreased at 20.00 and 24.00 in wt controls, but not in *β5*^−/−^ RPE/choroid. *Msr1* (SR-BI) did not indicate any variation in gene expression. Results are displayed as 2^ΔΔCt^ averages ± standard deviation in arbitrary units (a.u.), reference is wt at 8.00 (light onset), *n* = 3–6. Protein expression levels were augmented at 10.00 for MARCO (**A**) and at 7.00–8.00, 10.00 and 12.00–20.00 for CD36 (**B**) in wild-type samples (wt), while SR-AI (**A**), SR-BI and SR-B2/LIMP-2 (**B**) did not show any significant changes. Western blots for the nuclear pore complex protein (NPCP) and tubulin (Tub.) control proteins show no variation along the different hours of the light:dark cycle studied in wild-type mice, as indicated (**C**).

In vivo POS uptake by RPE cells follows a circadian rhythm, peaking 1.5–2 hours after light onset [2,5]. We have previously shown that β5 integrin and MerTK receptors both display a rhythmic expression pattern increasing after the phagocytic peak (Parinot et al., under revision). We thus assessed the expression profile of our five SRs along the light:dark cycle on control wild-type and β5 integrin knockout (*β5*^−/−^) mice in which the phagocytic rhythm was abolished (Figure 5) [5]. Class A *Marco* expression showed a peak of gene expression at 11 AM (11.00), 1 hour after the phagocytic peak in control mice, while the peak was observed at 7 (7.00) and 8AM (8.00) in *β5*^−/−^ mice just before light onset (Figure 5A). In contrast, *Msr1* [*SR-AI*] exhibited an arrhythmic gene expression pattern in control and knockout models. Class B *CD36* had an expression peak at 10 (10.00) and 11 AM (11.00) at the phagocytic peak, and just after in wild-type mice, while overall expression was lowered and at a steady state in *β5*^−/−^ animals (Figure 5B). Surprisingly, class B *Scarb1* [*SR-BI*] displayed a small expression rise at 9 (9.00) and 10 AM (10.00) at the time of the phagocytic peak in *β5*^−/−^ mice, and *Scarb2* [*SR-B2*/*LIMP-2*] expression declined at 8 PM (20.00), the time of the light offset, and midnight (24.00) in controls.

At the protein level, both MARCO and CD36 also displayed variations in their expression patterns (Figure 5A,B). While MARCO showed a burst of protein expression at the time of the phagocytic peak (Figure 5A), CD36 (Figure 5B) expression increase appeared to span over multiple time-points, i.e., before light onset (7.00–8.00), at the time of maximum phagocytosis (10.00), and from noon (12.00) to light offset (20.00). In contrast, the three other candidates showed steady-state expression levels along the light:dark cycle. The nuclear pore complex protein and tubulin controls validated the equal sample loading on our SDS-PAGE gels (Figure 5C).

## 3. Discussion

All receptors expressed in macrophages were detected in both RPE cell lines tested, rat RPE-J and human ARPE-19. Size differences between cell types were observed for some receptors (MARCO, CD36), which can be associated with tissue-specific splicing or differences in receptor glycosylation [50,51]. Yet, in vivo tissue expression was not equivalent between receptors: class A MARCO and class B SR-BI, especially in RPE/choroid fractions, appeared to show a more limited expression on immunoblots than CD36 and SR-B2/LIMP-2, which were detected at high levels, with SR-AI showing an intermediate expression pattern. Their respective expression levels were matched on histological sections, with limited expression levels of SR-AI, SR-BI and, to a lower extent, MARCO, while CD36 and SR-B2/LIMP-2 showed more widespread expression patterns, including in what appeared to be blood vessels, and higher expression levels. However, overall expression at the cell surface and function of receptors can be quite different characteristics, as some receptors such as MARCO, SR-AI and CD36 have been previously shown to become greatly overexpressed at the cell membrane upon cell stimulation with apoptotic cells, including via the limitation of autophagy in the cells [52,53]. Interestingly, ADAM17, already shown to cleave the MerTK internalization receptor to regulate its function, seems to participate in CD36 cleavage to regulate its surface expression levels, thus suggesting ADAM17 could target several receptors at the RPE cell surface [15,54]. We here confirm that the augmentation of the number of receptors available at the cell surface upon phagocytosis also takes place in RPE cells for CD36, as well as for the other SRs, even if at a more limited extent depending on the receptor.

In addition, our data underline the importance of the subcellular localization at the cell surface, especially with specific membrane subdomains such as lipid rafts known to concentrate receptors, cytoskeletal proteins, and signaling pathways [47,48,55]. CD36 has been associated with lipid rafts in macrophages [53], and we now confirm that it is also the case in RPE cells. We also show that all tested SRs do localize and/or relocate, totally or partially, within lipid rafts in RPE cells during POS phagocytosis. Localization and concentration in lipid raft subdomains suggest that all these receptors, either directly or indirectly, activate signaling platforms such as PI3K/Rac1, Grb2, p130Cas, Rab11a and Rab14, thus stimulating the actin cytoskeleton for pseudopod closure and internalization [37,54,56,57,58].

CD36 has been shown to be sufficient to confer the capacity to phagocytose apoptotic cells to non-macrophagic cells [59]. However, in the retina, the constant contact between POS and RPE cells renders a tight control of receptor activation and function crucial. Previously, CD36 has been suggested to regulate the speed of POS internalization [26]. In our blocking antibodies experiment, pre-incubation of the cells was necessary to replicate this feature, thus suggesting that CD36 receptors need to be inactivated before POS are in contact with them. This is consistent with the observation that CD36 receptors act as dimers or multimers [50,51], as blocking antibodies might be efficient only on isolated monomers. Pre-incubation was also a prerequisite for SR-B2/LIMP-2 antibody inhibition, suggesting these receptors might also work as multimers. Surprisingly, discrepancies were observed depending on the inhibition approach: while siRNA downregulation impacted both the amounts of tethered and engulfed POS for these two receptors, antibody inhibition had an effect on the dynamics of phagocytosis. This could be due to the difference between diminishing the overall quantities of available receptors that can reach the cell surface, and disturbing their function at the membrane where they are already inserted in macromolecular systems. Most importantly, both CD36 and SR-B2/LIMP-2 appeared to act in a similar fashion on controlling the phagocytic speed as their blockade accelerated phagocytosis. However, their timeline differed slightly, as SR-B2/LIMP-2 appeared to colocalize with POS and lipid rafts earlier than CD36. Interestingly, in vivo gene and protein expression was rhythmic only for CD36, implying that regulation or SR-B2/LIMP-2 function might not be linked to its expression levels, but maybe to other features such as receptor recycling or trafficking from intracellular storage areas. This receptor is much less studied, hence many mysteries about its regulation and function still remain. However, first known as a lysosomal-related protein [43], recent crystallography studies identified an intriguing characteristic: the binding of some ligands such as cholesterol foster receptors towards a dimeric state with a higher affinity for PtdSer [60]. In this context, our data suggest, for the first time, that SR-B2/LIMP-2 dimer receptors play active roles at the cell surface in the elimination of PtdSer-coated POS.

In contrast, the third receptor of the class B family, SR-BI, gave more puzzling results: it did not seem to associate directly with POS during phagocytosis, despite being mostly located in lipid rafts and making clusters at the cell surface upon POS challenge with other proteins recognized by WGA lectins. As well, while downregulation of its expression via siRNA transfection had no effect on POS phagocytosis, antibody inhibition without pre-incubation diminished both POS tethering and uptake, even though SR-BI has also been suggested to work as multimers/dimers [50,51]. SR-BI might thus act as an accessory receptor regulating the function of other receptors and indirectly influencing POS phagocytosis. This idea is supported by the fact that in integrin β5 knockout mice, in which the phagocytic peak is absent, expression of the SR-BI encoding gene (*Scarb1*) augments just before and at the time of the phagocytic peak, a potential compensatory feature from the cells.

Surprisingly, we observed that antibody blocking of MARCO did not have any significant impact on POS phagocytosis, while MARCO did associate with POS and lipid rafts and its gene and protein expression increased neatly just after the phagocytic peak. This suggests that MARCO did participate in POS phagocytosis, especially at 3 hours of in vitro phagocytosis—an intermediate step—, but its role could be taken over by another receptor when absent, with scavengers being known to take each other’s function in some instances. SR-AI, on the other hand, somewhat co-localized with POS, and was partially associated with lipid rafts as early as 1 hour after the start of POS challenge. However, siRNA inhibition reduced POS internalization while antibody inhibition stimulated internalization and maybe also binding, even if not significantly. This contrasting result, combined with the arrhythmic gene and protein expression profile, suggests a potential regulatory role in the early steps of POS elimination by RPE cells.

Taken together, our results add pieces to the RPE phagocytic machinery puzzle and show how well organized and controlled this critical function for vision is. Indeed, SRs appear to be involved in various steps of phagocytosis, either early for class A receptors MARCO and SR-AI, or more in the middle or at the end of the process for class B SR-B2/LIMP-2 and the MARCO. While some receptors such as CD36 and SR-B2/LIMP-2 are strongly associated with POS and their inhibition has a great impact on POS phagocytosis, others appear to either play an indirect role (SR-BI) or be used as accessory/redundant receptors (MARCO, SR-AI). Overall, the tight regulation of POS phagocytosis turns out to be based on a very complex machinery of receptors acting sequentially along different steps. First, MerTK receptors control the amounts of tethered POS that can be then engulfed via αvβ5 integrin receptors [10]. Second, αvβ5 integrin receptors and their ligands MFG-E8 start phagocytosis in a timely fashion via intracellular signaling pathways phosphorylating MerTK [5,9]. Third, other receptors, such as CD36 and now SR-B2/LIMP-2, control the speed of POS internalization [26]. In addition, besides these two late receptors, other SRs appear to play a more minor role, such as class A MARCO and SR-AI, or an indirect role such as class B SR-BI. Interestingly, among these scavenger receptors, some have been previously shown or suggested to act as co-receptors in other tissues such as MARCO for αvβ5 integrin [37] or SR-AI for MerTK [40], thus reinforcing the significance of our data.

Overall, there might be even more receptors implicated in regulating the phagocytic machinery in RPE cells, such as Toll-Like Receptors (TLRs) [61,62,63,64,65,66]. Moreover, the study of the retinal phenotype and of in vivo POS phagocytosis profiles of animal models devoid of these scavenger receptors will be of particular interest to definitely answer these remaining questions, and shed light on new pathological mechanisms and thus potential treatment targets not identified thus far.

## 4. Materials and Methods

### 4.1. Reagents, Antibodies and Cell Culture

Reagents were from Life Technologies (Courtaboeuf, France), unless otherwise stated. Antibodies used for the various experiments are detailed in Supplementary Table S1.

### 4.2. Cell Culture and siRNA transfection

The rat RPE-J cell line (ATCC) was maintained at 32 °C and 5% CO_2_ in DMEM with 4% CELLect Gold FCS (ICN) (both LGC Standards, Molsheim, France), supplemented with 10 mM HEPES and 1% non-essential amino acids (NEAAs). For immunocytochemistry (ICC) and phagocytosis quantification experiments, RPE-J cells were plated on Alcian blue-coated 24- or 96-well plates, respectively, and allowed to polarize for 6 days before use. When using siRNA silencing, cells were double transfected with rat ON-TARGETplus SMARTpool siRNAs (rat CD36 L-062017-00, rat Marco L-097276-02, rat Msr1 L-104326-02 [SR-AI], rat Scarb1 L-098018-01 [SR-BI], rat Scarb2 L-062021-01 [SR-B2]) at days 1 and 3 post-split using the DharmaFECT 4 siRNA Transfection Reagent as instructed (all from Dharmacon, Horizon Discovery Biosciences Limited, Cambridge, UK). Transfection efficiency was verified using the siGLO RISC-free Control siRNA (D-001600-01) and specificity by comparing with the ON-TARGETplus non-targeting Pool (L-001810-10). Phagocytosis assays were performed on day 4 post-split.

J774A.1 macrophages (ATCC) and the human ARPE-19 cell line (ATCC) were cultivated at 37 °C with 5% CO_2_ in DMEM containing 10% FCS and supplemented with 1% NEAAs and 1% sodium pyruvate, or in DMEM without sodium pyruvate containing 1% FCS, respectively.

### 4.3. Animals and Tissue Collection

Wild-type mice (129T2/SvEmsJ) were housed under cyclic 12-hour light/12-hour dark conditions (light onset at 8:00) and fed ad libitum. Animals were handled according to the Association for Research in Vision and Ophthalmology (ARVO) Statement for the Use of Animals in Ophthalmic and Vision Research, and protocols were approved by the Charles Darwin Animal Experimentation Ethics Committee from Sorbonne Université and the French Ministry for Education, Higher Studies and Research (APAFIS#1631-2015090415466433).

For experiments, mice were sacrificed by CO_2_ asphyxiation at 10.00 (rod phagocytosis peak) for immunoblots or different times during the day for the analysis of circadian expression. The 12 time points analysed along the light:dark cycle were: 4.00, 6.00, 7.00, 8.00 (light onset), 9.00, 10.00 (rod phagocytosis peak), 11.00, 12.00, 16.00, 20.00 (light offset), 22.00 and 24.00. Eyeballs were carefully enucleated and rinsed in HBSS without Ca^2+^ and Mg^2+^. The lens and vitreous humor were dissected out (“cup”). For some samples, retinas were delicately separated from the eyecups containing the RPE/choroid layers, and tissues were quickly frozen in liquid nitrogen. For each animal, one eye was used for RNA extraction and gene expression testing and the fellow eye for protein expression levels assessment (see corresponding sections below). For tissue sections, full eyeballs were immersed in Davidson fixative for 1 hour at 4 °C, a small window in the cornea was made and eyeballs were further fixed in Davidson for 3 hours at 4 °C. The anterior segment was dissected out above the iris and samples were placed in Davidson for 3 more hours at 4 °C. After overnight dehydration steps using the Spin Tissue Processor STP 120 (Myr, Fisher Scientific SAS, Illkirch, France), eyecups were embedded in paraffin and 5-μm sections were cut and deposited onto glass slides.

### 4.4. Sample Lysis and Immunoblotting

Cultured cells and tissues were solubilized in 50 mM HEPES, 150 mM NaCl, 10% glycerol, 1.5 mM MgCl_2_, and 1% Triton X-100 pH 7.4 buffer with 1 mM PMSF and 1% each of protease and phosphatase inhibitor cocktails, and sodium orthovanadate (Sigma-Aldrich, Saint Quentin Fallavier, France). Whole cell lysates—representing approximately 4–10% of a 10-cm culture dish (10–50 µg depending on the cell type) and 6–15% of a full eyecup or separated cup/retina from one eyecup (20–40 µg for Figure 1, 12 µg for Figure 5)—were separated on SDS-polyacrylamide gels and electroblotted onto nitrocellulose membranes (Whatman, VWR, Rosny-sous-Bois, France). Immunoblots were probed with primary antibodies (Supplementary Table S1) overnight at 4 °C and secondary antibodies coupled with horseradish peroxydase for 2 hours, with 4 washes in 1X TBS 1% Tween-20 at RT after each incubation, followed by chemiluminescence detection (PerkinElmer SAS, Courtaboeuf, France). Chemiluminescence films (Amersham, Dutscher SAS, Bernolsheim, France) were scanned, and images were processed using the Adobe Photoshop CS6 version 13.0 software (Adobe Systems Incorporated, San Jose, CA, USA).

### 4.5. POS Isolation

POS were isolated according to a well-established protocol [67] from porcine eyes obtained fresh from the slaughterhouse. Briefly, retinae were retrieved from eyeballs dissected in the dark under dim red light and collected in homogenization buffer (20% sucrose, 20 mM tris acetate pH 7.2, 2 mM MgCl_2_, 10 mM glucose, 5 mM taurine). After thorough shaking and gauze filtering, retina suspensions were separated on continuous 25–60% sucrose gradients (in tris acetate pH 7.2, 10 mM glucose, 5 mM taurine) and centrifuged at 25,000 rpm for 50 min at 4 °C (Beckman SW 32 Ti swinging rotor). After collection, orange bands containing POS were sequentially diluted and washed in a series of 3 solutions (20 mM tris acetate pH 7.2, 5 mM taurine; 10% sucrose, 20 mM tris acetate pH 7.2, 5 mM; 10% sucrose, 20 mM tris acetate pH 7.2, 5 mM taurine) associated with centrifugation steps at 5000 rpm for 10 min at 4 °C (Beckman JA25.50 rotor). After resuspension and counting, aliquots of POS stocks were frozen at −80 °C in DMEM containing 2.5% sucrose.

Fluorescence labeling of POS was performed with 1 mg/mL fluorescein isothiocyanate (FITC) (Molecular Probes, Fisher Scientific SAS, Illkirch, France) for 2 hours at room temperature (RT) on a rotator in 10% sucrose, 20 mM sodium phosphate pH 7.2, and 5 mM taurine. Labeled POS were then washed, counted, and frozen as described above.

### 4.6. POS Phagocytosis

Cells were challenged with around 10 POS per RPE cell resuspended in DMEM for 1, 3 and 5 hours (immunocytochemistry, raft immunoblots) or with 10 FITC-POS for 1.5 or 3 hours for phagocytosis quantification. In some assays, anti-receptor antibodies (Supplementary Table S1) at 1 µg/mL were added to FITC-POS in DMEM for a 3-hour phagocytic challenge, with or without a 1-hour pre-incubation step with antibodies alone in DMEM. For POS phagocytosis quantification assays, cells were washed three times with PBS-CM (0.2 mM Ca^2+^, 1 mM Mg^2+^) at the end of the incubation times. To measure internalized POS, some wells were incubated with trypan blue for 10 min to quench the fluorescence of surface-bound FITC-labeled POS, as previously described [68]. Non-treated wells allowed the measurement of total phagocytosis, corresponding to the fluorescence of both bound and internalized POS. All cells were then washed twice with PBS-CM and fixed with ice-cold methanol. Nuclei were counterstained using DAPI. FITC-POS and DAPI-labeled nuclei were quantified by fluorescence plate reading (Infinite M1000, Magellan version 6 software, Tecan France SASU, Lyon, France). Binding ratios were calculated by subtracting results obtained in internalization (trypan blue-treated) from total phagocytosis (untreated) wells. Corresponding standard deviations (s.d.) were calculated using the following formula: s.d._binding_ = √((s.d._total_^2^/n_total_) + (s.d._intern_^2^/n_intern_)).

### 4.7. Immunohistochemistry, Immunocytochemistry and Microscopy

Five-micrometre section slides around the optic nerve area (for consistency between samples) were used. Paraffin was removed using the SafeSolv solvent substitute (Q Path, VWR, Rosny-sous-Bois, France) for 30 min, followed by sequential baths of 100% ethanol for 30 min, followed by 90% and 70% ethanol for 10 min each. Antibodies sites were unmasked by cooling down the slides in warm 1X citrate buffer 1X ddH_2_O for 20 min on ice, and RPE pigments were removed in a 5% H_2_O_2_, 1X SSC solution containing deionized formamide under illumination for 20 min. After membranes permeabilization with 0.3% Triton in 1X TBS for 5 min, non-specific signals were blocked by using 4% BSA, 4% donkey serum, 100 mM glycine in 1X TBS. Sections were stained overnight at 4 °C with anti-receptor antibodies (Supplementary Table S1) or anti-goat and anti-rabbit IgG controls. Appropriate AlexaFluor488 secondary antibodies (Molecular Probes) diluted 1:1000 in 1% BSA, 1X TBS were incubated on sections for 30 min. Nuclei were counterstained with DAPI, and slides were mounted using the Vectashield medium (Vector Laboratories, Burlingame, CA, USA).

After incubating cells with unlabeled POS resuspended in DMEM for 1, 3 or 5 hours, cells were washed twice with ice-cold HBSS-CM (0.2 mM Ca^2+^, 1 mM Mg^2+^) or PBS-CM for surface or intracellular labeling, respectively. Briefly, for live labeling of cell surface proteins, antibodies were diluted in HBSS-CM and cells were incubated for 45 min on ice. Cells were washed twice with ice-cold HBSS-CM before either TCA fixation on ice for 15 min and re-hydration with 30 mM glycine in PBS-CM for 5 min thrice at RT (CD36, MARCO, SR-B2/LIMP-2), or ice-cold methanol fixation for 15 min followed by re-hydration in PBS-CM for 10 min twice at RT (SR-BI), or 4% PFA fixation in PBS-CM for 15 min at RT and quenching in 50 mM NH_4_Cl for 15 min (SR-AI). Next, non-specific sites were blocked with 1% BSA in PBS-CM. Primary antibody incubation for intracellular labeling were performed overnight at 4 °C or for 2 h at RT. Cells were washed 3 times with PBS-CM then with 1% BSA in PBS-CM. AlexaFluor secondary antibodies (Molecular Probes) were incubated on cells for 1–2 hours at RT, followed by washing with PBS-CM, labeling cell nuclei with DAPI and mounting onto glass slides with FluoroMount-G Mounting Medium (Interchim).

All fluorescent images were acquired with an upright Olympus FV1000 confocal microscope using the Fluoview version 2.1c software (Olympus, Rungis, France). Equivalent stacks of images were compiled for each series and further treated equally for signal output levels using NIH ImageJ (version 1.53o, https://imagej.nih.gov/ij/, last accessed on 31 January 2022).

### 4.8. Lipid Rafts Isolation by a Detergent-Free Method

RPE cells cultivated in 24-well plates were pelleted (195 g for 5 min at 4 °C) after different times of POS challenge (1, 3 and 5 hours) and resuspended in 1.34 mL of 0.5 M sodium carbonate, pH 11.5, with protease inhibitor cocktail and phosphatase inhibitor cocktail 1, 2 and 3 (Sigma-Aldrich, Saint Quentin Fallavier, France) [52]. The homogenate was sheared through a 26-gauge needle with 10 complete passes, then sonicated with 3 10-s bursts. The homogenate was adjusted to 40% sucrose by adding 2.06 mL of 60% sucrose in MBS (25 mM MES, pH 6.4, 150 mM NaCl, 250 mM sodium carbonate), placed under a 5–30% discontinuous sucrose gradient, and centrifuged at 34,000 rpm for 15–18 h at 4 °C (Beckman SW 41 Ti swinging rotor). Nine 1.24 mL fractions were harvested from the top of the tube, mixed with 9 volumes of MBS and centrifuged at 40,000 rpm for 1 h at 4 °C (Beckman SW 41 Ti rotor). Supernatants were discarded, and membrane pellets were resuspended in 100 µL of 1% SDS in MBS. When required (low expression levels: SR-AI, CD36), samples were concentrated on concentration columns (Amicon Ultra Centrifugal Filters 10K, Millipore SAS, Molsheim, France).

### 4.9. Quantification of Gene Expression

RNA were extracted from separated retina and RPE/Choroid according to the manufacturer’s protocol using 2 DNAse steps (Illustra RNAspin Mini, GE Healthcare, Vélizy-Villacoublay, France). RNAs were verified on 1% agarose gels and yields assessed using a spectrophotometer. Then, 500 ng of RNAs were converted to cDNAs in a 50-µL volume following the instructions provided for 1 hour at 42 °C (Reverse Transcription System, Promega, Charbonnières, France). qPCR reactions using the SYBR Green PCR Master Mix were processed as follows on a 7500 Fast Real-Time PCR System apparatus (both Applied Biosystems) using the primer pairs detailed in Supplementary Table S2: 50 °C for 2 min, 95 °C for 10 min, followed by 40 cycles of 95 °C for 15 sec, 60 °C for 1 min. The *ribosomal protein Rho0* (*Rplp0*) gene was used as internal control. Oligonucleotides were designed in order to obtain 150-bp amplicons and associated melting curves from a typical experiment series are pictured in Supplementary Figure S1. Relative amounts of each target gene were calculated using the 2^−^^ΔΔ^^Ct^ method and amounts at 8 AM (8.00, light onset) were set as 1.

### 4.10. Statistical Analysis

All experiments were repeated between 3 and 9 times. The statistical significance of results was determined using the Bonferroni–Dunn method for multiple comparisons, and each row was analysed individually without assuming a consistent s.d. Significance thresholds of adjusted *p* values were set as follows: * *p* < 0.05, ** *p* < 0.01, *** *p* < 0.001, **** *p* < 0.0001.

## Supplementary Materials

The following supporting information can be downloaded at: https://www.mdpi.com/article/10.3390/ijms23073445/s1.

## References

[1] Strauss O. (2005). The retinal pigment epithelium in visual function. Physiol. Rev..

[2] LaVail M.M. (1976). Rod outer segment disk shedding in rat retina: Relationship to cyclic lighting. Science.

[3] Bok D., Hall M.O. (1971). The role of the pigment epithelium in the etiology of inherited retinal dystrophy in the rat. J. Cell Biol..

[4] Nandrot E., Dufour E.M., Provost A.C., Péquignot M.O., Bonnel S., Gogat K., Marchant D., Rouillac C., Sépulchre de Condé B., Bihoreau M.T. (2000). Homozygous deletion in the coding sequence of the *c-mer* gene in RCS rats unravels general mechanisms of physiological cell adhesion and apoptosis. Neurobiol. Dis..

[5] Nandrot E.F., Kim Y., Brodie S.E., Huang X., Sheppard D., Finnemann S.C. (2004). Loss of synchronized retinal phagocytosis and age-related blindness in mice lacking alphavbeta5 integrin. J. Exp. Med..

[6] Gal A., Li Y., Thompson D.A., Weir J., Orth U., Jacobson S.G., Apfelstedt-Sylla E., Vollrath D. (2000). Mutations in *MERTK*, the human orthologue of the RCS rat retinal dystrophy gene, cause retinitis pigmentosa. Nat. Genet..

[7] Parinot C., Nandrot E.F. (2016). A Comprehensive Review of Mutations in the *MERTK* Proto-Oncogene. Adv. Exp. Med. Biol..

[8] Finnemann S.C., Rodriguez-Boulan E. (1999). Macrophage and retinal pigment epithelium phagocytosis: Apoptotic cells and photoreceptors compete for alphavbeta3 and alphavbeta5 integrins, and protein kinase C regulates alphavbeta5 binding and cytoskeletal linkage. J. Exp. Med..

[9] Nandrot E.F., Anand M., Almeida D., Atabai K., Sheppard D., Finnemann S.C. (2007). Essential role for MFG-E8 as ligand for alphavbeta5 integrin in diurnal retinal phagocytosis. Proc. Natl. Acad. Sci. USA.

[10] Nandrot E.F., Silva K.E., Scelfo C., Finnemann S.C. (2012). Retinal pigment epithelial cells use a MerTK-dependent mechanism to limit the phagocytic particle binding activity of αvβ5 integrin. Biol. Cell.

[11] Fadok V.A., Savill J.S., Haslett C., Bratton D.L., Doherty D.E., Campbell P.A., Henson P.M. (1992). Different populations of macrophages use either the vitronectin receptor or the phosphatidylserine receptor to recognize and remove apoptotic cells. J. Immunol..

[12] Ruggiero L., Connor M.P., Chen J., Langen R., Finnemann S.C. (2012). Diurnal, localized exposure of phosphatidylserine by rod outer segment tips in wild-type but not *Itgb5^−/−^* or *Mfge8^−/−^* mouse retina. Proc. Natl. Acad. Sci. USA.

[13] Hanayama R., Tanaka M., Miwa K., Shinohara A., Iwamatsu A., Nagata S. (2002). Identification of a factor that links apoptotic cells to phagocytes. Nature.

[14] Nakano T., Kawamoto K., Higashino K., Arita H. (1996). Prevention of growth arrest-induced cell death of vascular smooth muscle cells by a product of growth arrest-specific gene, gas6. FEBS Lett..

[15] Law A.L., Parinot C., Chatagnon J., Gravez B., Sahel J.A., Bhattacharya S.S., Nandrot E.F. (2015). Cleavage of Mer tyrosine kinase (MerTK) from the cell surface contributes to the regulation of retinal phagocytosis. J. Biol. Chem..

[16] Chang Y., Finnemann S.C. (2007). Tetraspanin CD81 is required for the alphavbeta5-integrin-dependent particle-binding step of RPE phagocytosis. J. Cell Sci..

[17] Ravichandran K.S. (2011). Beginnings of a good apoptotic meal: The find-me and eat-me signaling pathways. Immunity.

[18] Prabhudas M., Bowdish D., Drickamer K., Febbraio M., Herz J., Kobzik L., Krieger M., Loike J., Means T.K., Moestrup S.K. (2014). Standardizing scavenger receptor nomenclature. J. Immunol..

[19] Alquraini A., El Khoury J. (2020). Scavenger receptors. Curr. Biol..

[20] Plüddemann A., Neyen C., Gordon S. (2007). Macrophage scavenger receptors and host-derived ligands. Methods.

[21] Ryeom S.W., Silverstein R.L., Scotto A., Sparrow J.R. (1996). Binding of anionic phospholipids to retinal pigment epithelium may be mediated by the scavenger receptor CD36. J. Biol. Chem..

[22] Tait J.F., Smith C. (1999). Phosphatidylserine receptors: Role of CD36 in binding of anionic phospholipid vesicles to monocytic cells. J. Biol. Chem..

[23] Greenberg M.E., Sun M., Zhang R., Febbraio M., Silverstein R., Hazen S.L. (2006). Oxidized phosphatidylserine-CD36 interactions play an essential role in macrophage-dependent phagocytosis of apoptotic cells. J. Exp. Med..

[24] Murphy J.E., Tacon D., Tedbury P.R., Hadden J.M., Knowling S., Sawamura T., Peckham M., Phillips S.E., Walker J.H., Ponnambalam S. (2006). LOX-1 scavenger receptor mediates calcium-dependent recognition of phosphatidylserine and apoptotic cells. Biochem. J..

[25] Ryeom S.W., Sparrow J.R., Silverstein R.L. (1996). CD36 participates in the phagocytosis of rod outer segments by retinal pigment epithelium. J. Cell Sci..

[26] Finnemann S.C., Silverstein R.L. (2001). Differential roles of CD36 and alphavbeta5 integrin in photoreceptor phagocytosis by the retinal pigment epithelium. J. Exp. Med..

[27] Sparrow J.R., Ryeom S.W., Abumrad N.A., Ibrahimi A., Silverstein R.L. (1997). CD36 expression is altered in retinal pigment epithelial cells of the RCS rat. Exp. Eye Res..

[28] Sun M., Finnemann S.C., Febbraio M., Shan L., Annangudi S.P., Podrez E.A., Hoppe G., Darrow R., Organisciak D.T., Salomon R.G. (2006). Light-induced oxidation of photoreceptor outer segment phospholipids generates ligands for CD36-mediated phagocytosis by retinal pigment epithelium: A potential mechanism for modulating outer segment phagocytosis under oxidant stress conditions. J. Biol. Chem..

[29] Courtois Y. (2010). The role of CD36 receptor in the phagocytosis of oxidized lipids and AMD. Aging.

[30] Rigotti A., Acton S.L., Krieger M. (1995). The class B scavenger receptors SR-BI and CD36 are receptors for anionic phospholipids. J. Biol. Chem..

[31] Picard E., Houssier M., Bujold K., Sapieha P., Lubell W., Dorfman A., Racine J., Hardy P., Febbraio M., Lachapelle P. (2010). CD36 plays an important role in the clearance of oxLDL and associated age-dependent sub-retinal deposits. Aging.

[32] Dufour E.M., Nandrot E., Marchant D., Van Den Berghe L., Gadin S., Issilame M., Dufier J.L., Marsac C., Carper D., Menasche M. (2003). Identification of novel genes and altered signaling pathways in the retinal pigment epithelium during the Royal College of Surgeons rat retinal degeneration. Neurobiol. Dis..

[33] Duncan K.G., Bailey K.R., Kane J.P., Schwartz D.M. (2002). Human retinal pigment epithelial cells express scavenger receptors BI and BII. Biochem. Biophys. Res. Commun..

[34] Provost A.C., Péquignot M.O., Sainton K.M., Gadin S., Sallé S., Marchant D., Hales D.B., Abitbol M. (2003). Expression of SR-BI receptor and StAR protein in rat ocular tissues. C. R. Biol..

[35] Duncan K.G., Hosseini K., Bailey K.R., Yang H., Lowe R.J., Matthes M.T., Kane J.P., LaVail M.M., Schwartz D.M., Duncan J.L. (2009). Expression of reverse cholesterol transport proteins ATP-binding cassette A1 (ABCA1) and scavenger receptor BI (SR-BI) in the retina and retinal pigment epithelium. Br. J. Ophthalmol..

[36] Provost A.C., Vede L., Bigot K., Keller N., Tailleux A., Jaïs J.P., Savoldelli M., Ameqrane I., Lacassagne E., Legeais J.M. (2009). Morphologic and electroretinographic phenotype of SR-BI knockout mice after a long-term atherogenic diet. Invest. Ophthalmol. Vis. Sci..

[37] Xing Q., Feng Y., Sun H., Yang S., Sun T., Guo X., Ji F., Wu B., Zhou D. (2021). Scavenger receptor MARCO contributes to macrophage phagocytosis and clearance of tumor cells. Exp. Cell Res..

[38] Platt N., Suzuki H., Kurihara Y., Kodama T., Gordon S. (1996). Role for the class A macrophage scavenger receptor in the phagocytosis of apoptotic thymocytes in vitro. Proc. Natl. Acad. Sci. USA.

[39] Cao W.M., Murao K., Imachi H., Sato M., Nakano T., Kodama T., Sasaguri Y., Wong N.C., Takahara J., Ishida T. (2001). Phosphatidylinositol 3-OH kinase-Akt/protein kinase B pathway mediates Gas6 induction of scavenger receptor a in immortalized human vascular smooth muscle cell line. Arterioscler. Thromb. Vasc. Biol..

[40] Todt J.C., Hu B., Curtis J.L. (2008). The scavenger receptor SR-A I/II (CD204) signals via the receptor tyrosine kinase Mertk during apoptotic cell uptake by murine macrophages. J. Leukoc. Biol..

[41] Araki N., Higashi T., Mori T., Shibayama R., Kawabe Y., Kodama T., Takahashi K., Shichiri M., Horiuchi S. (1995). Macrophage scavenger receptor mediates the endocytic uptake and degradation of advanced glycation end products of the Maillard reaction. Eur. J. Biochem..

[42] Platt N., Suzuki H., Kodama T., Gordon S. (2000). Apoptotic thymocyte clearance in scavenger receptor class A-deficient mice is apparently normal. J. Immunol..

[43] Heybrock S., Kanerva K., Meng Y., Ing C., Liang A., Xiong Z.J., Weng X., Ah Kim Y., Collins R., Trimble W. (2019). Lysosomal integral membrane protein-2 (LIMP-2/SCARB2) is involved in lysosomal cholesterol export. Nat. Commun..

[44] Neculai D., Schwake M., Ravichandran M., Zunke F., Collins R.F., Peters J., Neculai M., Plumb J., Loppnau P., Pizarro J.C. (2013). Structure of LIMP-2 provides functional insights with implications for SR-BI and CD36. Nature.

[45] Carrasco-Marín E., Fernández-Prieto L., Rodriguez-Del Rio E., Madrazo-Toca F., Reinheckel T., Saftig P., Alvarez-Dominguez C. (2011). LIMP-2 links late phagosomal trafficking with the onset of the innate immune response to Listeria monocytogenes: A role in macrophage activation. J. Biol. Chem..

[46] Gamp A.C., Tanaka Y., Lüllmann-Rauch R., Wittke D., D’Hooge R., De Deyn P.P., Moser T., Maier H., Hartmann D., Reiss K. (2003). LIMP-2/LGP85 deficiency causes ureteric pelvic junction obstruction, deafness and peripheral neuropathy in mice. Hum. Mol. Genet..

[47] Pike L.J. (2003). Lipid rafts: Bringing order to chaos. J. Lipid Res..

[48] Head B.P., Patel H.H., Insel P.A. (2014). Interaction of membrane/lipid rafts with the cytoskeleton: Impact on signaling and function: Membrane/lipid rafts, mediators of cytoskeletal arrangement and cell signaling. Biochim. Biophys. Acta.

[49] El Biari K., Gaudioso A., Fernández-Alonso M.C., Jiménez-Barbero J., Cañada F.J. (2019). Peptidoglycan Recognition by Wheat Germ Agglutinin. A View by NMR. Nat. Prod. Comm..

[50] Thorne R.F., Meldrum C.J., Harris S.J., Dorahy D.J., Shafren D.R., Berndt M.C., Burns G.F., Gibson P.G. (1997). CD36 forms covalently associated dimers and multimers in platelets and transfected COS-7 cells. Biochem. Biophys. Res. Commun..

[51] Reaven E., Cortez Y., Leers-Sucheta S., Nomoto A., Azhar S. (2004). Dimerization of the scavenger receptor class B type I: Formation, function, and localization in diverse cells and tissues. J. Lipid Res..

[52] Bonilla D.L., Bhattacharya A., Sha Y., Xu Y., Xiang Q., Kan A., Jagannath C., Komatsu M., Eissa N.T. (2013). Autophagy regulates phagocytosis by modulating the expression of scavenger receptors. Immunity.

[53] Ferracini M., Rios F.J., Pecenin M., Jancar S. (2013). Clearance of apoptotic cells by macrophages induces regulatory phenotype and involves stimulation of CD36 and platelet-activating factor receptor. Mediat. Inflamm..

[54] Jiang C., Liu Z., Hu R., Bo L., Minshall R.D., Malik A.B., Hu G. (2017). Inactivation of Rab11a GTPase in Macrophages Facilitates Phagocytosis of Apoptotic Neutrophils. J. Immunol..

[55] Averaimo S., Assali A., Ros O., Couvet S., Zagar Y., Genescu I., Rebsam A., Nicol X. (2016). A plasma membrane microdomain compartmentalizes ephrin-generated cAMP signals to prune developing retinal axon arbors. Nat. Commun..

[56] Stuart L.M., Bell S.A., Stewart C.R., Silver J.M., Richard J., Goss J.L., Tseng A.A., Zhang A., Khoury J.B.E., Moore K.J. (2007). CD36 signals to the actin cytoskeleton and regulates microglial migration via a p130Cas complex. J. Biol. Chem..

[57] Tao H., Yancey P.G., Babaev V.R., Blakemore J.L., Zhang Y., Ding L., Fazio S., Linton M.F. (2015). Macrophage SR-BI mediates efferocytosis via Src/PI3K/Rac1 signaling and reduces atherosclerotic lesion necrosis. J. Lipid Res..

[58] Seixas E., Escrevente C., Seabra M.C., Barral D.C. (2018). Rab GTPase regulation of bacteria and protozoa phagocytosis occurs through the modulation of phagocytic receptor surface expression. Sci. Rep..

[59] Ren Y., Silverstein R.L., Allen J., Savill J. (1995). CD36 gene transfer confers capacity for phagocytosis of cells undergoing apoptosis. J. Exp. Med..

[60] Conrad K.S., Cheng T.W., Ysselstein D., Heybrock S., Hoth L.R., Chrunyk B.A., Am Ende C.W., Krainc D., Schwake M., Saftig P. (2017). Lysosomal integral membrane protein-2 as a phospholipid receptor revealed by biophysical and cellular studies. Nat. Commun..

[61] Sun B., Qi N., Shang T., Wu H., Deng T., Han D. (2010). Sertoli cell-initiated testicular innate immune response through toll-like receptor-3 activation is negatively regulated by Tyro3, Axl, and mer receptors. Endocrinology.

[62] Stewart C.R., Stuart L.M., Wilkinson K., van Gils J.M., Deng J., Halle A., Rayner K.J., Boyer L., Zhong R., Frazier W.A. (2010). CD36 ligands promote sterile inflammation through assembly of a Toll-like receptor 4 and 6 heterodimer. Nat. Immunol..

[63] Mukhopadhyay S., Varin A., Chen Y., Liu B., Tryggvason K., Gordon S. (2011). SR-A/MARCO-mediated ligand delivery enhances intracellular TLR and NLR function, but ligand scavenging from cell surface limits TLR4 response to pathogens. Blood.

[64] Sigola L.B., Fuentes A.-L., Millis L.M., Vapenik J., Murira A. (2016). Effects of Toll-like receptor ligands on RAW 264.7 macrophage morphology and zymosan phagocytosis. Tissue Cell.

[65] Cao D., Luo J., Chen D., Xu H., Shi H., Jing X., Zang W. (2016). CD36 regulates lipopolysaccharide-induced signaling pathways and mediates the internalization of Escherichia coli in cooperation with TLR4 in goat mammary gland epithelial cells. Sci. Rep..

[66] Yamada C., Beron-Pelusso C., Algazzaz N., Heidari A., Luz D., Rawas-Qalaji M., Toderas I., Mascarenhas A.K., Kawai T., Movila A. (2016). Age-dependent effect between MARCO and TLR4 on PMMA particle phagocytosis by macrophages. J. Cell Mol. Med..

[67] Parinot C., Rieu Q., Chatagnon J., Finnemann S.C., Nandrot E.F. (2014). Large-scale purification of porcine or bovine photoreceptor outer segments for phagocytosis assays on retinal pigment epithelial cells. J. Vis. Exp..

[68] Finnemann S.C., Bonilha V.L., Marmorstein A.D., Rodriguez-Boulan E. (1997). Phagocytosis of rod outer segments by retinal pigment epithelial cells requires alpha(v)beta5 integrin for binding but not for internalization. Proc. Natl. Acad. Sci. USA.

