# Supplementary material for: Pleiotropic Roles of Scavenger Receptors in Circadian Retinal Phagocytosis: A New Function for Lysosomal SR-B2/LIMP-2 at the RPE Cell Surface
Source: Int J Mol Sci. 2022 Mar 22;23(7):3445. doi: 10.3390/ijms23073445 (PMC8998831; doi:10.3390/ijms23073445)
Supplement: Supplementary file 1 [file ijms-23-03445-s001.zip › ijms-1601396-supplementary-update.pdf]

International Journal of *Molecular Sciences*

MDPI

Supplementary Materials

Supplementary Table S1. Name, reference, species and dilutions corresponding to the antibodies used in our various experiments as indicated. Monoclonal antibodies are specified as mAb and clone numbers are indicated in brackets, when available. ROS: Rod outer segments. Antibodies used for ICC on live cells before fixation are diluted 1:50. * Antibodies used for blocking experiments.

Antibody	Reference/s	Species	ICC	IHC	WB	Rafts WB
Actin	Abcam ab3280 [ACTN05 (C4)]	mouse mAb	—	—	—	1:100
Caveolin	BD Transduction Laboratories 610060	rabbit	1:1000	—	—	—
	Abcam ab2910	rabbit	—	—	—	1:500
CD36	R&D Systems AF2519 *	goat	1:50	—	1:800	—
	Novus Biologicals NB400-144	rabbit	—	1:200	—	1:500
Ezrin	Sigma E8897 [3C12]	mouse mAb	1:500	—	—	—
Flotillin-1	BD Transduction Laboratories 610820	mouse mAb	1:200	—	—	1:1,000
MARCO	R&D Systems AF2956	goat	1:50	—	1:500	—
	Biorbyt orb6345 *	rabbit	—	1:200	—	—
Rhodopsin	Millipore MAB5316 [RET-P1]	mouse mAb	1:300	—	—	—
ROS	kind gift from Michael Hall	rabbit ascites	1:300	—	—	—
SR-AI	R&D Systems AF1797 *	goat	1:50	1:200	—	—
	Abcam ab151707 [EPR7536]	rabbit mAb	—	—	1:800	1:250
SR-BI	Abcam ab52629 [EP1556Y]	rabbit mAb	1:50	1:200	1:500	1:500
	Novus Biologicals NB400-101 *	rabbit	—	—	—	—
SR-B2/LIMP-2	R&D Systems AF1888 *	goat	1:50	1:200	1:3,000	1:3,000
WGA-FITC	Sigma L4895	—	1:250	—	—	—
ZO-1	Invitrogen 61-7300	rabbit	1:300	—	—	—
	eBioscience 14-9776 [R26.4C]	rat mAb	1:300	—	—	—

Supplementary Table S2. Mouse gene names and corresponding forward (F) and reverse (R) primer sequences.

Gene		5′-3′ Sequence
Mouse *CD36*	F	CAAAGAGGTCCTTACACATAC
	R	TGTGAAGTTGTCATCCTCTGT
Mouse *Marco*	F	GTCAGCAGTTCAACAACCTC
	R	CCTTTTCTCCCTTCTCAGCA
Mouse *Msr1 [SR-AI]*	F	TGAACGAGAGGATGCTGACTG
	R	GGAGGGGCCATTTTTAGTGC
Mouse *Scarb1 [SR-BI]*	F	CAGAATGTCAGCACCTGCAG
	R	GTGACCGGATGGATGTCTAG
Mouse *Scarb2 [SR-B2/LIMP-2]*	F	TTACCAAGCCGACGAGAAGT
	R	ACCAAGCCAAAGAACACACC
Mouse *RPLP0*	F	CCTGAAGTGCTCGACATCAC
	R	TGCCAGGACGCGCTTGTAC

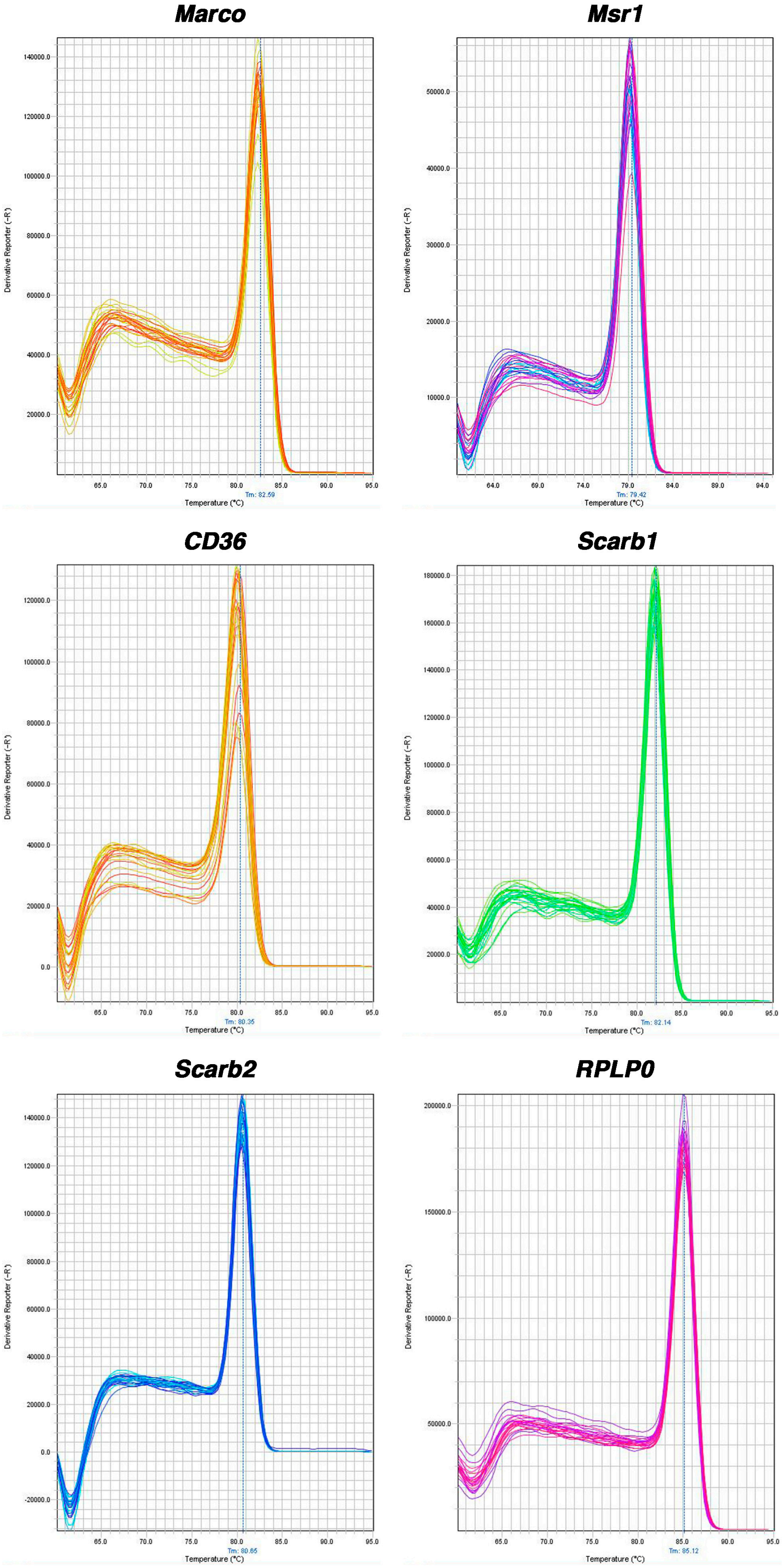

Supplementary Figure S1. Typical melting curves of qPCR primer pairs for each gene as indicated.
